# Tract‐Specific White Matter Hyperintensities Disrupt Brain Networks and Associated With Cognitive Impairment in Mild Traumatic Brain Injury

**DOI:** 10.1002/hbm.70050

**Published:** 2024-11-29

**Authors:** Xuan Li, Zhuonan Wang, Haonan Zhang, Wenpu Zhao, Qiuyu Ji, Xiang Zhang, Xiaoyan Jia, Guanghui Bai, Yizhen Pan, Tingting Wu, Bo Yin, Lei Shi, Zhiqi Li, Jierui Ding, Jie Zhang, David H. Salat, Lijun Bai

**Affiliations:** ^1^ The Key Laboratory of Biomedical Information Engineering, Ministry of Education, Department of Biomedical Engineering, School of Life Science and Technology Xi'an Jiaotong University Xi'an China; ^2^ PET‐CT Center, The First Affiliated Hospital of Xi'an Jiaotong University Xi'an China; ^3^ Department of Radiology The Second Affiliated Hospital and Yuying Children's Hospital of Wenzhou Medical University Wenzhou China; ^4^ Department of Neurosurgery The Second Affiliated Hospital and Yuying Children's Hospital of Wenzhou Medical University Wenzhou China; ^5^ Department of Clinical Laboratory Shuguang Hospital Affiliated to Shanghai University of Chinese Traditional Medicine Shanghai China; ^6^ Department of Radiation Medicine, School of Preventive Medicine Air Force Medical University Xi'an China; ^7^ Athinoula A. Martinos Center for Biomedical Imaging, Department of Radiology Massachusetts General Hospital Charlestown Massachusetts USA

**Keywords:** cognitive impairment, dynamic, mild traumatic brain injury, tract‐specific, white matter hyperintensities

## Abstract

Traumatic brain injury (TBI) is considered to initiate cerebrovascular pathology, involving in the development of multiple forms of neurodegeneration. However, it is unknown the relationships between imaging marker of cerebrovascular injury (white matter hyperintensity, WMH), its load on white matter tract and disrupted brain dynamics with cognitive function in mild TBI (mTBI). MRI data and neuropsychological assessments were collected from 85 mTBI patients and 52 healthy controls. Between‐group difference was conducted for the tract‐specific WMH volumes, white matter integrity, and dynamic brain connectivity (i.e., fractional occupancies [%], dwell times [seconds], and state transitions). Regression analysis was used to examine associations between white matter damage, brain dynamics, and cognitive function. Increased WMH volumes induced by mTBI within the thalamic radiation and corpus callosum were highest among all tract fibers, and related with altered fractional anisotropy (FA) within the same tracts. Clustering identified two brain states, segregated state characterized by the sparse inter‐independent component connections, and default mode network (DMN)‐centered integrated state with strongly internetwork connections between DMN and other networks. In mTBI, higher WMH loads contributed to the longer dwell time and larger fractional occupancies in DMN‐centered integrated state. Every 1 mL increase in WMH volume within the left thalamic radiation was associated with a 47% increase fractional occupancies, and contributed to 65.6 s delay in completion of cognitive processing speed test. Our study provided the first evidence for the structural determinants (i.e., small vessel lesions) that mediate the spatiotemporal brain dynamics to cognitive impairments in mTBI.

## Graphical abstract

Increased WMH lesion loads within the thalamic radiation and corpus callosum were highest among all tract fibers following mTBI, and related with altered FA within the same tracts. Higher WMH burden contributed to the longer dwell time and larger fractional occupancies in DMN‐centered integrated state in mTBI.

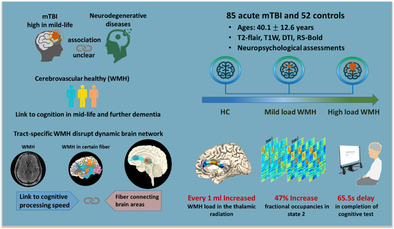

## 1 Introduction

Traumatic brain injury (TBI) poses a significant concern for the millions of populations injured annually and stands as one of the strongest environmental risk factors for the onset of neurodegenerative diseases such as late‐onset Alzheimer's disease (AD) (Graham and Sharp 2019; Livingston et al. 2020; Johnson et al. 2023). Recent research indicates that TBI‐induced cerebrovascular injuries may accelerate amyloid β production, tau hyperphosphorylation, and perivascular accumulation which typically observed in both AD and postmortem brains of TBI (Ramos‐Cejudo et al. 2018; Zysk et al. 2019; Johnson et al. 2023). Cerebrovascular injury is recognized as a significant contributor to worsening cognitive deficits in AD and white matter hyperintensities (WMH) being its primary biomarker in imaging (Taylor et al. 2017; Garnier‐Crussard et al. 2022, 2023). Currently, it is widely accepted that the diffuse axonal injury (DAI) is a substantial pathophysiology component of brain injury, which is related to microhemorrhage during the acute phase, and is highly related to the patient outcome (Sharp, Scott, and Leech 2014). Postmortem imaging and histology evidence demonstrate that mild TBI (mTBI), constituting over 80% of all TBI (Levin and Diaz‐Arrastia 2015; Silverberg et al. 2020), results in the WMH co‐localization with iron‐laden macrophages, indicating a potential origin of cerebrovascular injury as the underlying mechanism (Griffin et al. 2019). Despite previous work demonstrated that WMH have been identified in mTBI, the contribution of WMH was still unclear.

Previous research has demonstrated that higher volume of WMH are linked to an increased risk of AD (Taylor et al. 2017) and impaired resting brain functional connectivity (Zhang et al. 2022). However, these studies typically utilized global measures of WMH volume averaged across the entire white matter or large brain regions, without considering the anatomical boundaries of fiber tracts (Langen et al. 2018; Tuladhar et al. 2020). In addition, recent studies into ischemic WMH have highlighted the importance of lesion location and the anatomical connections between WMH and cortical gray matter (Taylor et al. 2017; Vergoossen et al. 2021). Emerging studies show that WMH disrupts the long‐range white matter tract and thereby affects the underlying structural connectivity within the large‐scale brain network (Tuladhar et al. 2020); subsequently, the affected structural connection disrupts functional connectivity, impairing information transfer across distal brain regions and producing cognitive deficits (Jolly et al. 2020). Therefore, there is a pressing need to further understand how WMH functions in mTBI, the precise patterns by which white matter connectivity is disrupted, and consequently, the functional brain networks that are predicted to be affected.

Besides that, considering that the interactions among large‐scale brain systems are highly nonstationary, this functional connectivity based on time‐averaged or static connectivity provides limited information about the functional organization of brain circuits (van der Horn et al. 2020). Recently, our study demonstrated that dynamic, but not static, time‐varying cross‐network interactions among the triple‐network, are a sensitive, robust, and clinically meaningful biomarkers in identifying multiple cognitive functions in mTBI (Li et al. 2023). Therefore, it is necessary to unravel the contribution of WMH to dynamic functional networks and its mediated link by white matter connection provided a thorough understanding of both the brain biological configuration and WMH‐related cognitive impairments following mTBI. We used the automated fiber‐tract quantification (AFQ), considering the regional intra‐tract tissue characteristics, to test whether WMH disrupts tracts fibers in the regional but not along entire (Yeatman et al. 2012). Moreover, we used dynamic functional connection analysis to test whether the disrupted whiter matter tract fiber would interrupt the dynamic communications between the tract‐connected brain areas.

## 2 Materials and Methods

### 2.1 Participants

A total of 85 patients with mTBI (45 male, ages of 40.1 ± 12.6 years) and 52 matched healthy controls (HC) (22 male, ages of 37.3 ± 12.5 years) were included in the study. Inclusion and exclusion criteria for mTBI patients was reported in our previous studies (Niu et al. 2019). In detail, patients with mild traumatic brain injury (mTBI) were selected with the following criteria: (a) patients with normal CT findings (Glasgow Coma Score of 13–15); (b) one or more of the following: confusion or disorientation, post‐traumatic amnesia for less than 24 h, loss of consciousness for 30 min or less, and/or other transient neurological abnormalities such as focal signs, seizure, and intracranial lesion not requiring surgery; (c) diagnosed within 1 week after onset of mTBI; (d) 18 years or older. The exclusion criteria included: (a) a history of a previous brain injury, neurological disease, long‐term psychiatric history, or a history of concurrent substance or alcohol abuse; (b) a structural abnormality in neuroimaging (CT and MRI); (c) intubation and/or skull fracture, and administration of sedatives; (d) the manifestation of mTBI caused by medications, alcohol, and drugs for other injuries (such as systemic injuries, facial injuries, or intubation); (e) other problems (such as psychological trauma, language impairment, or coexisting medical conditions); (f) caused by penetrating craniocerebral injury. Screening for mTBI was based on the World Health Organization's Collaborating Centre for Neurotrauma Task Force (Holm et al. 2005). All participants provided written informed consent, and all research procedures were approved by the Ethics Committee of The School of Life Science and Technology in Xi'an Jiaotong University and conducted following the Declaration of Helsinki.

### 2.2 Neuropsychological Assessments

Comprehensive neuropsychological assessments were conducted for both mTBI patients and HC. All of patients were free of litigation to avoid any bias on the testing performance. The neuropsychological measures included: (i) the Digital Symbol Coding score (DSC) of Wechsler Adult Intelligence Scale (WAIS)‐III and Trail‐Making Test Part A (TMT‐A) to measure cognitive information processing speed (IPS) (Arnett and Labovitz 1995); (ii) the Forward Digit Span (FDS) of WAIS‐III to assess executive functions (Sun et al. 2005); (iii) the Backward Digit Span (BDS) of WAIS‐III to assess working memory (Sun et al. 2005); (iv) the Verbal Fluency (VF) Test to examine language ability and semantic memory (Joy, Kaplan, and Fein 2004).

### 2.3 Imaging Acquisition

The protocol for scanning included a non‐contrast CT scan for acute head injury. Individuals underwent a multimodality MRI scan (3.0 T, GE750) with a 32‐channel head coil. The scan parameters of High‐resolution T1‐weighted three‐dimensional Magnetization Prepared Rapid Gradient Echo (MPRAGE) sequence were as follows: repetition time (TR) = 8.15 ms; echo time (TE) = 3.17 ms; slice thickness = 1 mm; flip angle (FA) = 9; field of view (FOV) = 256 mm × 256 mm; matrix size = 256 × 256. The scan parameters of T2‐weighted fluid‐attenuated inversion recovery (FLAIR) sequence were as follows: TR = 9000 ms; TE = 95 ms; FA = 150; thickness = 5 mm; slices = 20; FOV = 240 mm × 240 mm; matrix size = 173 × 256. The scan parameters of diffusion‐weighted imaging represent as follows: TR = 7300 ms; TE = 99 ms; slice thickness = 3 mm; flip angle = 90; slices = 50; FOV = 256 mm × 256 mm; matrix size = 128 × 128; two averages; voxel size = 2 mm × 2 mm ×3 mm. Diffusion tensor imaging (DTI) scan (*b* = 1000 s/mm^2^) were acquired with 30 diffusion gradient orientations and the *b* = 0 repeated two times. Resting‐state fMRI data were acquired using a gradient‐recalled echo planar imaging sequence with a total 180 volumes of 54 slices covering the whole brain, and the scan parameters were as follows: TR = 2500 ms; TE = 30 ms; slice thickness = 3 mm; flip angle = 90; FOV = 216 mm × 216 mm; matrix size = 64 × 64; voxel size = 3 mm × 3 mm × 3 mm. During scanning, all participants were instructed to relax, close their eyes, keep awake, and try not to think of anything in particular.

### 2.4 DTI Preprocessing and Automated Fiber‐Tract Quantification

Preprocessing of DTI data was performed using FMRIB Software Library (FSL; https://fsl.fmrib.ox.ac.uk/fsl) software (Jenkinson et al. 2012). First, non‐brain tissues were removed from the DTI data using the brain extraction tool algorithm in FSL. Next, eddy correction was carried out to correct the effects of head movement and geometric distortion caused by the eddy current. Diffusion data were visually inspected after each processing step to control obvious artifacts. The diffusion metric FA sensitively reflected the microstructural integrity of white matter fibers, which was calculated using the DTIFIT tool in FSL. Each participant's FA image was then aligned to MNI‐152 standard space using non‐linear registration tool (FNIRT).

Then, we used a MATLAB‐based open source software AFQ (https://github.com/jyeatman/AFQ) to identify 20 fiber tracts in the individual's brain (Yeatman et al. 2012). AFQ can compute the diffusion metrics at multiple nodes along a white matter pathway (tract) instead of merely a global mean value, which is more sensitive to focal/regional variability. A brief description of the primary steps was listed here (details listed in the Data S1): (i) whole brain tractography used a deterministic streamlines tracking algorithm (STT; Basser et al. 2000); (ii) fiber tract segmentation was performed with the waypoint ROI procedure (Wakana et al. 2007); (iii) fiber refinement was accomplished by comparing each candidate fiber to fiber tract probability maps (Hua et al. 2008); (iv) each fiber was sampled to 100 equidistant nodes that can be used to compute diffusion metric value at each node along the fiber. The identified 20 fiber tracts included the bilateral thalamic radiation, corticospinal tract, cingulum cingulate, cingulum hippocampus, inferior front‐occipital fasciculus, inferior longitudinal fasciculus, superior longitudinal fasciculus, uncinate fasciculus, arcuate fasciculus, posterior and anterior corpus callosum.

### 2.5 WMH Segmentation and Projection Onto Fiber Tracts

T2‐weighted FLAIR images were processed and analyzed using the FMRIB Software Library (FSL; https://fsl.fmrib.ox.ac.uk/fsl) software (Griffanti et al. 2016). In detail, the FLAIR images were normalized to the Montreal Neurologic Institute (MNI) space. Then, WMH was segmented from the whole brain FLAIR images by using the brain intensity abnormality classification algorithm (BIANCA). As a fully automated, supervised method for WMH detection, BIANCA classifies the image's voxels based on their intensity and spatial features, and the output image represents the probability per voxel of being WMH. The optimal threshold value was set as 0.8 according to previous studies (Williamson et al. 2018; Schlemm et al. 2022).

Tract‐specific WMH load were then calculated by superimposing the spatially normalized WMH maps onto the Johns Hopkins University International Consortium for Brain Mapping probabilistic fiber tract atlas (JHU‐ICBM‐tracts) in the MNI152 standard space (Hua et al. 2008). The atlas was comprised of probability maps for 20 main fiber tracts and the AFQ also used same atlas to evaluate similarity for candidate fiber and standard fiber. To calculate the WMH volume for a specific fiber tract, the WMH map was overlaid on the corresponding probability map and used as a mask. The voxel‐wise probabilities were then summed up across all voxels within this mask. Finally, the tract‐specific WMH volumes were normalized to the tracts’ total volumes, calculated as the sum of voxel‐wise fiber tract probabilities (from the JHU‐ICBM‐tracts) throughout the whole brain.

### 2.6 Dynamic Connectivity Quantification

A standard functional MRI preprocessing procedure was implemented using DPABI (http://rfmri.org/dpabi) and SPM 12 (https://www.fil.ion.ucl.ac.uk/spm), including the slice‐timing correction, realignment, normalization (normalized to the MNI space), spatial smoothing (6 mm smoothing kernel), regression of nuisance variables (24 motion parameters, white matter, global and cerebrospinal fluid [CSF] signals), and band‐pass filtering (0.01 Hz < *f* < 0.1 Hz). Participants with motion greater than 2 mm or 2° rotation in any direction during the scanning were excluded. Considering that the dynamic functional connection analysis may be sensitive to gross head motion effects, we also calculated the mean frame‐wise displacement (FD) to scrubbed the “bad” volumes with excessive head motion (FD > 1 mm; Li et al. 2023).

The dynamic functional network connectivity was calculated using a sliding‐window correlation approach, and the procedures were also consistent with our previous study (Li et al. 2023). For each subject, the optimal window length was determined as 50 TRs and shifted with a step size of 1 TR (i.e., 2 s) each time, resulting in 126 windows in total. In each sliding window, the time courses of each pair of the 227 ROIs were used to calculate functional connectivity and a 227 × 227 correlation matrix was obtained (Fiorenzato et al. 2019). In addition, we performed other window length (30 and 70 TR) and shifting step (1 TR) to further examine the possible effects on dynamic functional connectivity results (details in the Data S1). Then, we adopted a *k*‐means clustering algorithm on windowed functional connectivity matrices to assess the reoccurring functional connectivity patterns (states), as expressed by the frequency and structure of these states (Li et al. 2023). For the temporal properties of dynamic functional connectivity (DFC) states, we computed three measures in each subject, such as: (i) mean dwell time in each state, measured by the average number of consecutive windows in the same state; (ii) fractional of time spent in each state, computed as the proportion of all windows in each state; (iii) total number of transitions, defined as the probability of transitioning from one state to another state (details in the Data S1).

### 2.7 Serum Inflammation Examination

We also collected the serum samples from both patients and HC to test whether the inflammation levels can affect the brain white matter integrity. The 9‐plex panel of serum cytokines included the interleukin (IL)‐1β, IL‐4, IL‐6, IL‐8, IL‐10, IL‐12, chemokine ligand 2 (CCL2), interferon‐γ (IFN‐γ), and tumor necrosis factor‐α (TNF‐α). The details were reported in our previous study (Sun et al. 2019).

### 2.8 Statistical Analysis

Between‐group comparison of the demographic characteristics, neuropsychological assessments, and post‐concussion symptom were assessed using an independent two‐sample *t*‐test for continuous variables and chi‐square test for categorical variables in the SPSS 25.0 software package (https://www.ibm.com/cn‐zh/analytics/spss‐statisticssoftware), with the statistical significance of *p* < 0.05. The Shapiro–Wilk W test was used to evaluate the normality of continuous variables. Between‐group comparison of WMH volume and DTI metrics were performed using the two‐sample *t*‐tests with the significance of *p* < 0.05 (false discovery rate [FDR] corrected). For the temporal properties of the DFC, between‐group differences in the mean dwell time, fractional of time spent, and total number of transitions were examined between HC and mTBI patients using a two‐sample *t*‐tests (*p* < 0.05, FDR corrected). One‐way analysis of variance (ANOVA) and post hoc analysis were used to compare the differences between more than two groups, with the Bonferroni correction for the multiple comparison correction. Generalized linear regression models were used to assess the association between WMH volume and cognitive performance, and temporal properties of dynamic functional connectivity states. This model was adjusted for nonmodifiable factors including age, sex, and education. *p* values were reported, with adjustments made for multiple comparison using the FDR method and statistical significance set as *p* < 0.05.

## 3 Results

### 3.1 Sample Characteristics

Patients with mTBI did not differ from HC regarding to the age and gender (Table 1). Patients had lower education level compared to HC (mTBI: 8.0 ± 4.16 years, HC: 10.8 ± 5.95; *p* = 0.001; Figure S1). Education level was then used as a regressor in the subsequent analysis. Compared to HC, patients presented worse performance in multiple cognitive domains (i.e., cognitive processing speed, executive function, working memory, verbal fluency; all for *p* < 0.05, FDR corrected). In addition, the HC individuals and two mTBI patient subgroups (sWMH and mWMH) did not differ with respect to age and sex distribution (see Altered WMH Volumes and in White Matter Tract). Two subgroups (sWMH and mWMH) had lower education level compared to HC. Therefore, education level was used as a regressor in the subsequent analysis. Compared with HC, sWMH presented worse performance in almost cognitive domain test (all *p* < 0.05, Bonferroni corrected), but the cognitive domain tests were not different between the mWMH and HC. Compared with mWMH, sWMH presented worse performance in IPS (*p* < 0.05, Bonferroni corrected), but the other cognitive tests were not different between the sWMH and mWMH (Table 2). For mTBI patients, the causes of injury included the road traffic accident (58.8%), incidental fall (15.3%), violence or assault (22.4%), and other mechanisms (3.5%).

**TABLE 1:** Demographic, neuropsychological, and symptom data for mTBI and HC.

Characteristics	mTBI (*n* = 85)	HC (*n* = 52)	*p* value
Age, years	40.07 (12.60)	37.27 (12.54)	0.208
Female/male	40/45	30/22	0.230
Education, years	8.0 (4.16)	10.80 (5.95)	0.001
Trauma mechanism			
Road traffic accident	50 (58.8%)		
Incidental fall	13 (15.3%)		
Violence or assault	19 (22.4%)		
Other mechanism	3 (3.5%)		
Cognitive and clinical assessments			
TMT‐A	71.72 (49.78)	44.94 (26.09)	< 0.001
DSC	30.98 (15.27)	45.04 (17.06)	< 0.001
FDS	7.51 (1.48)	8.23 (1.60)	0.008
BDS	3.56 (1.38)	4.27 (1.65)	0.008
VF	15.95 (4.99)	18.67 (5.97)	0.005

*Note:* Two sample *t*‐test was run to test between‐group differences (mTBI and HC). Chi‐square test was used for categorical variables. Continuous variables are expressed as mean (standard deviation) and categorical variables are expressed as frequency.

Abbreviations: BDS, backward digit span; FDS, forward digit span; HC, healthy controls; mTBI, mild traumatic brain injury; TMT‐A, trail making test A; VF, verbal fluency.

**TABLE 2:** Demographic, neuropsychological, and symptom data for sWMH, mWMH and HC.

Characteristics	mTBI (sWMH) (*n* = 28)	mTBI (mWMH) (*n* = 57)	HC (*n* = 52)	ANOVA	Post hoc		
				*p* value	*p*1 value	*p*2 value	*p*3 value
Age, years	43.32 (11.79)	38.47 (12.78)	37.27 (12.54)	0.112			
Female/male	48/61	23/18	36/29	0.343			
Education, years	5.82 (3.89)	9.07 (3.89)	10.8 (5.95)	< 0.001			
Cognitive and clinical assessments							
TMT‐A	96.03 (68.75)	59.77 (31.55)	44.94 (26.09)	< 0.001	< 0.001	< 0.001	0.172
DSC	24.11 (13.96)	34.35 (14.85)	45.04 (17.06)	< 0.001	0.015	< 0.001	0.001
FDS	7.07 (1.49)	7.72 (1.44)	8.23 (1.60)	0.005	0.197	0.004	0.240
BDS	3.43 (1.32)	3.63 (1.41)	4.27 (1.65)	0.025	0.99	0.052	0.081
VF	14.64 (4.90)	16.59 (4.96)	18.67 (5.97)	0.006	0.349	0.005	0.136

*Note:* One‐way ANOVA was run to test between‐group differences (sWMH, mWHM and HC). Chi‐square test was used for categorical variables. Continuous variables are expressed as mean (standard deviation) and categorical variables are expressed as frequency. *p*1 represents the post hoc *p* values from one‐way ANOVA between sWMH and mWMH. *p*2 represents the post hoc *p* values from one‐way ANOVA between sWMH and HC. *p*3 represents the post hoc *p* values from one‐way ANOVA between mWMH and HC.

Abbreviations: BDS, Backward digit span; DSC, digit symbol coding; FDS, Forward digit span; HC, healthy controls; mTBI, mild traumatic brain injury; mWMH, mild WMH load group; sWMH, severe WMH load group; TMT‐A, trail making test A; VF, verbal fluency.

### 3.2 Altered WMH Volumes and in White Matter Tract

**FIGURE 1:**
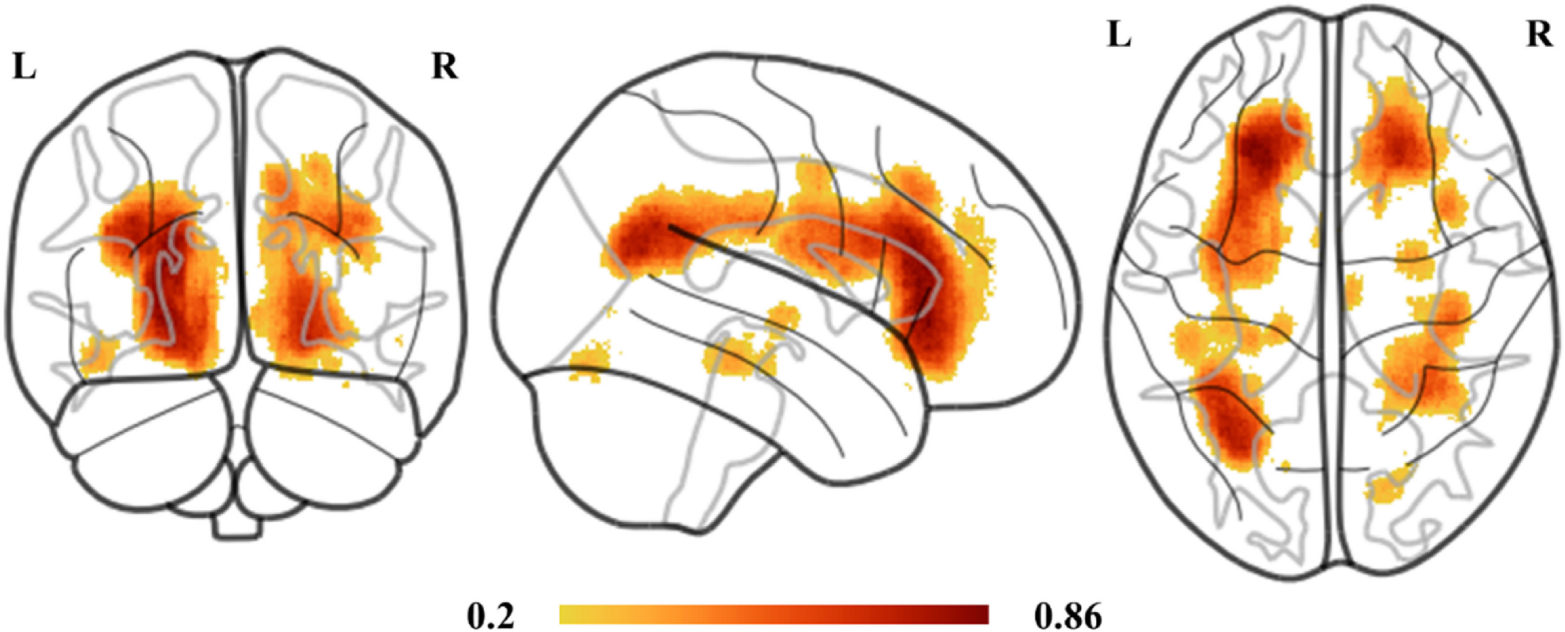
WMH probability map of all mTBI participants. The color bar represents probability values. mTBI, mild traumatic brain injury, WMH, white matter hyperintensities.

**FIGURE 2:**
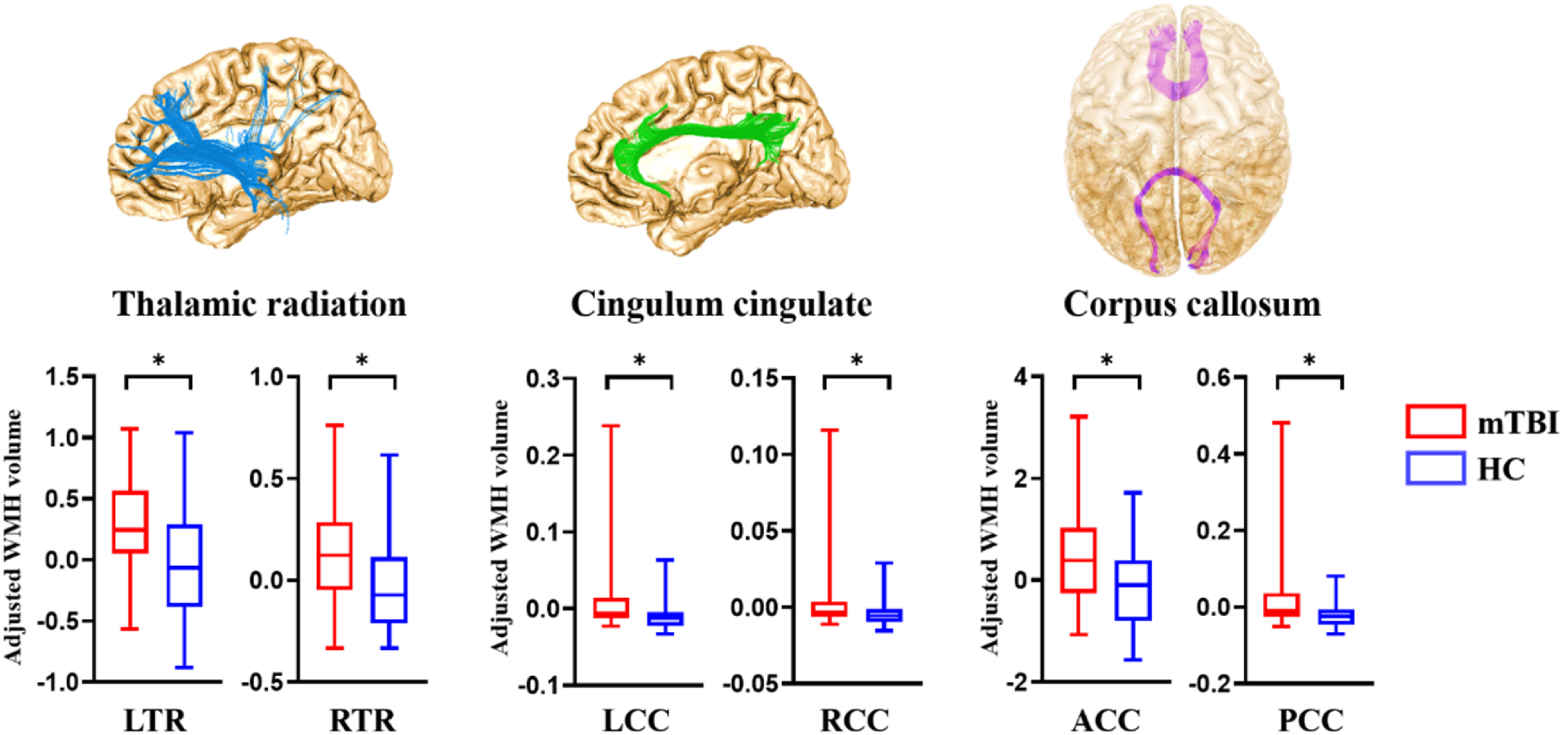
Group differences of WMH for white matter tract fiber between patients with mTBI and HC individuals. ACC, anterior corpus callosum; HC, healthy controls; LCC, left cingulum cingulate; LTR, left thalamic radiation; mTBI, mild traumatic brain injury; PCC, posterior corpus callosum; RCC, right cingulum cingulate; RTR, right thalamic radiation; WMH, white matter hyperintensities. * indicated a significance of *p* < 0.05, FDR corrected.

MRI scanning for mTBI patients was evaluated within 7 days post‐injury (2.8 ± 1.4 post‐injury days). WMH probability map of all the 85 participants was shown in the Figure 1. Compared with HC, tract‐specific WMH volumes were significantly increased in the patients with mTBI, primarily located within the bilateral thalamic radiation and cingulum cingulate, posterior and anterior part of the corpus callosum (*p* < 0.02, FDR corrected; Figure 2 and Table 3). Median of the total lesion burden for all these six fiber tracts was 1.89 mL (interquartile range [IQR], 1.12–2.73 mL). As for the ration of WMH lesion volume to each white matter tract, the most tract‐specific lesion burdens located in the left thalamic radiation (median 7.25%; IQR: 4.17%–9.56%), anterior corpus callosum (median 4.93%; IQR 2.15%–9.14%), and right thalamic radiation (median 3.66%; IQR: 1.46%–5.69%). According to the WMH lesion burden within these six tracts, mTBI patients were then divided into the mild WMH (mWMH) and severe WMH subgroups (sWMH). In detail, we calculated the standardized scores for WMH in the six white mater tracts by regressing out age, sex, and education in HC group. Then, patients with mTBI who scored 1.5 standard deviations above healthy control subjects on at least two white mater tracts were categorized as sWMH subgroup and the remaining as the mWMH subgroup (Li et al. 2023; Pan et al. 2023; Fiorenzato et al. 2019).

**TABLE 3:** Difference WMH load in 20 white matter fiber tracts between mTBI and HC.

Fibers	WMH (ml)		WMH load ratio (%)		Adjusted *p* value
	mTBI	HC	mTBI	HC	
LTR	0.598 (0.326)	0.425 (0.287)	6.92	4.91	< 0.001*
RTR	0.304 (0.218)	0.225 (0.184)	3.84	2.83	< 0.001*
LCST	0.011 (0.025)	0.008 (0.016)	0.19	0.15	0.24
RCST	0.002 (0.007)	0.0005 (0.001)	0.04	0.01	0.11
LCC	0.026 (0.052)	0.006 (0.015)	1.44	0.32	< 0.001*
RCC	0.0012 (0.027)	0.003 (0.006)	1.31	0.32	0.004*
LCH	0.00	0.00	—	—	—
RCH	0.0005 (0.001)	0.0007 (0.003)	0.06	0.09	0.72
ACC	0.048 (0.086)	0.013 (0.025)	6.01	3.92	< 0.001*
PCC	1.156 (0.924)	0.763 (0.487)	0.75	0.21	< 0.001*
LIFOF	0.401 (0.213)	0.379 (0.206)	6.93	6.51	0.017
RIFOF	0.241 (0.162)	0.235 (0.137)	3.52	3.43	0.06
LILF	0.113 (0.109)	0.112 (0.122)	1.91	1.91	0.25
RILF	0.008 (0.028)	0.004 (0.010)	0.22	0.12	0.23
LSLF	0.411 (0.293)	0.394 (0.244)	4.21	4.02	0.076
RSLF	0.399 (0.379)	0.521 (0.497)	5.12	6.73	0.87
LU	0.023 (0.021)	0.027 (0.019)	1.91	2.23	0.56
RU	0.011 (0.011)	0.011 (0.012)	1.51	1.33	0.082
LA	0.0006 (0.001)	0.0005 (0.0009)	0.73	0.61	0.38
RA	0.0008 (0.002)	0.001 (0.002)	0.26	0.35	0.88

*Note:* Two sample *t*‐test was run to test between‐group differences in WMH volume (mTBI and HC).

Abbreviations: ACC, anterior corpus callosum; LAF, left arcuate fasciculus; LCC, left cingulum cingulate; LCH, left cingulum hippocampus; LCST, left corticospinal tract; LIFOF, left inferior fronto‐occipital fasciculus; LILF, left inferior longitudinal fasciculus; LSLF, left superior longitudinal fasciculus; LTR, left thalamic radiation; LUF, left uncinate fasciculus; PCC, posterior corpus callosum; RAF, right arcuate fasciculus; RCC, right cingulum cingulate; RCH, right cingulum hippocampus; RCST, right corticospinal tract; RIFOF, right inferior fronto‐occipital fasciculus; RILF, right inferior longitudinal fasciculus; RSLF, right superior longitudinal fasciculus; RTR, right thalamic radiation; RUF, right uncinate fasciculus.

* Indicated a significance of *p* < 0.05, FDR corrected.

### 3.3 WMH Lesion Load Differences in Tract Profile of FA

**FIGURE 3:**
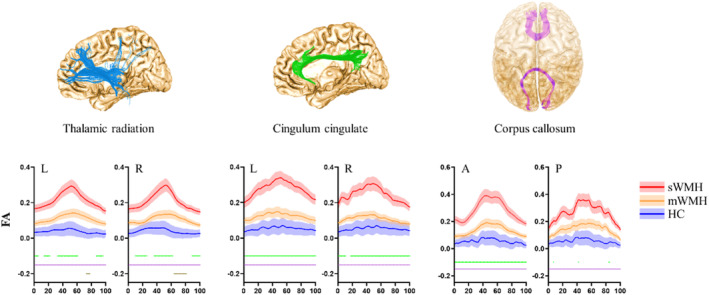
The pointwise comparison of FA profile among sWMH subgroup, mWMH subgroup and HC individuals. The green bars under the FA profile indicate the regions of significant difference between sWMH and mWMH individuals. The purple bars under the FA profile indicate the regions of significant difference between sWMH and HC individuals. The brown bars under the FA profile indicate the regions of significant difference between mWMH and HC individuals. The *x*‐axis represents the location between the beginning and termination waypoint regions of interest. mTBI, mild traumatic brain injury; WMH, white matter hyperintensities; sWMH, severe WMH load group; mWMH, mild WMH load group; HC, healthy controls; FA, fractional anisotropy; L, left; R, right; A, anterior; P, posterior.

In pointwise comparison of FA profiles, one‐way ANOVA analysis showed that altered FA in the bilateral thalamic radiation and cingulum cingulate, as well as the posterior and anterior corpus callosum. Post hoc analysis found that mTBI with sWMH had significantly increased FA compared with both the mWMH subgroup and HCs, mainly located in the entire bilateral thalamic radiation and cingulum cingulate, as well as the anterior portion of the corpus callosum. Of which, mTBI with mWMH subgroup exhibited the increased FA only in the anterior portion of the left thalamic radiation, compared with HC. There was no significant difference of FA in other white matter tracts (Figure 3).

### 3.4 Severe Inflammatory Response in mTBI

One‐way ANOVA analysis showed that altered serum inflammation cytokine levels of IL‐1β, IL‐6, and TNF‐α. Post hoc analysis found that the serum inflammation cytokine levels of IL‐1β, IL‐6, and TNF‐α were significant increase in sWMH group compared with HC, and the mWMH group showed increase serum inflammation cytokine levels of IL‐6 relative to HC. Between‐group difference was no significant in serum inflammation cytokine levels for the sWMH and mWMH subgroups (details in Tables S1 and S2).

### 3.5 Dynamic Functional Network Analysis

**FIGURE 4:**
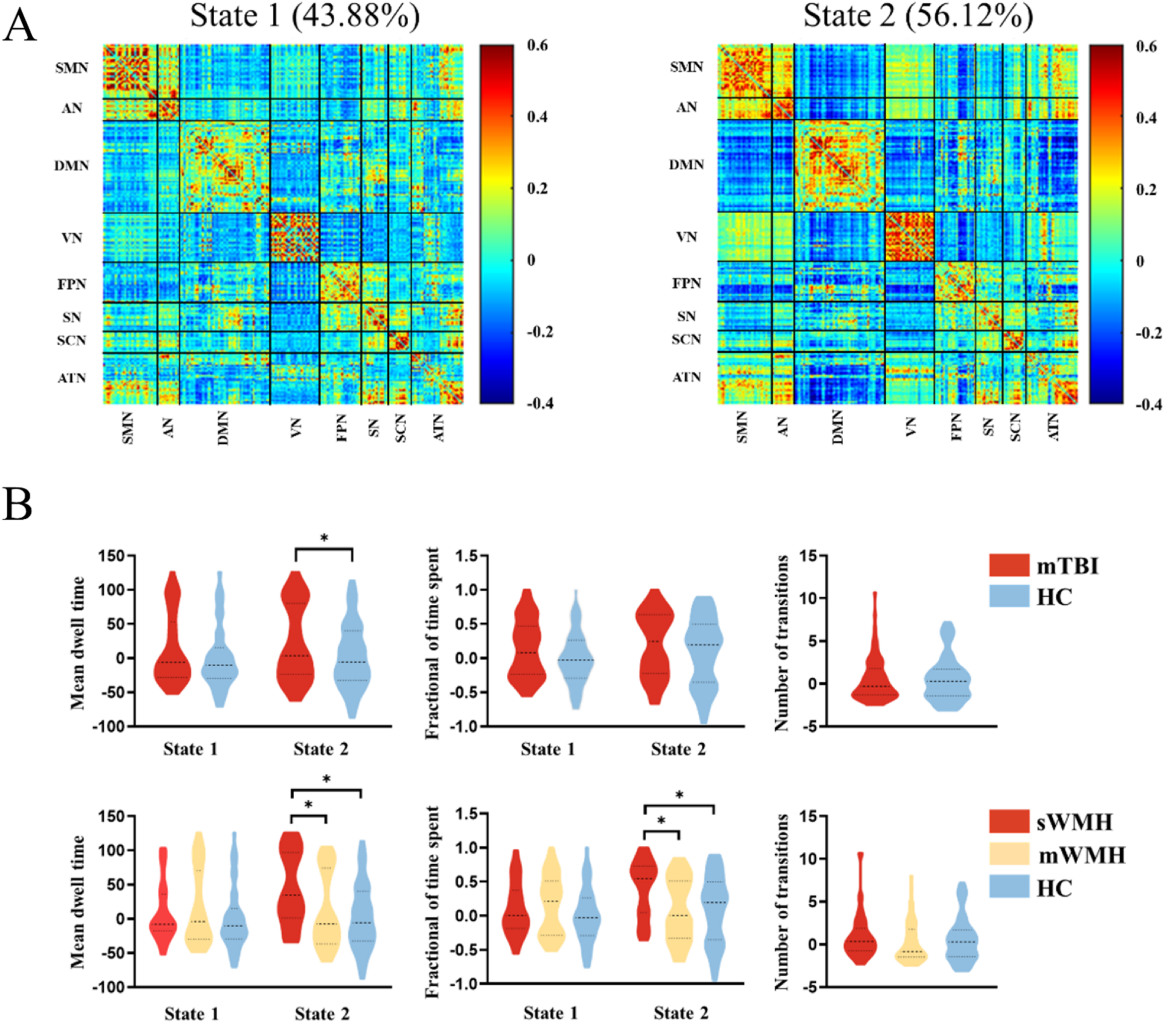
Dynamic functional connectivity state analysis. (A) Results of the clustering analysis per state cluster centroids for each state. The percentage of total occurrences are listed above each cluster median. (B) Temporal properties of dynamic FC states for the mTBI and HC groups and temporal properties of dynamic FC states for the sWMH subgroup, mWMH subgroup and HC groups. * indicated a significance of *p* < 0.05, FDR corrected. HC, healthy controls; mTBI, mild traumatic brain injury; WMH, white matter hyperintensities; sWMH, severe WMH load group; mWMH, mild WMH load group.

Based on the silhouette criterion of cluster validity index, the optimal number of clusters was determined as *k* = 2. In addition, the optimal number of clusters *k* was 2 by estimated Calinski–Harabasz coefficients and Davies–Bouldin coefficients on all windows of all subjects varying *k* from 2 to 6 (Li et al. 2023). We identified two functional connectivity states and the corresponding visualized connectivity patterns (centroids of clusters) were showed in the Figure 4A. State 1 was characterized by the sparse inter‐independent component connections, segregated state. While stronger internetwork connections were primarily observed in the state 2, involving the auditory network (AN), somatosensory and motor network (SMN), attention network (ATN), and default mode network (DMN)‐centered integrated state. Patients with higher WMH burden (sWMH subgroup) exhibited longer mean dwell time (*p* = 0.002, Bonferroni corrected) and spent more fractional of time (*p* = 0.02, Bonferroni corrected) only in the state 2, compared to patients with less WMH burden (mWMH subgroup). In addition, patients sWMH subgroup exhibited longer the mean dwell time (*p* = 0.004, Bonferroni corrected) and spent more fractional of time (*p* = 0.02, Bonferroni corrected) compared with HC (Figure 4B). No significant difference was detected between mWMH subgroup and HC (all *p* > 0.05).

### 3.6 Associations Between WMH Burden, Brain Dynamics, and Cognitive Performance

**FIGURE 5:**
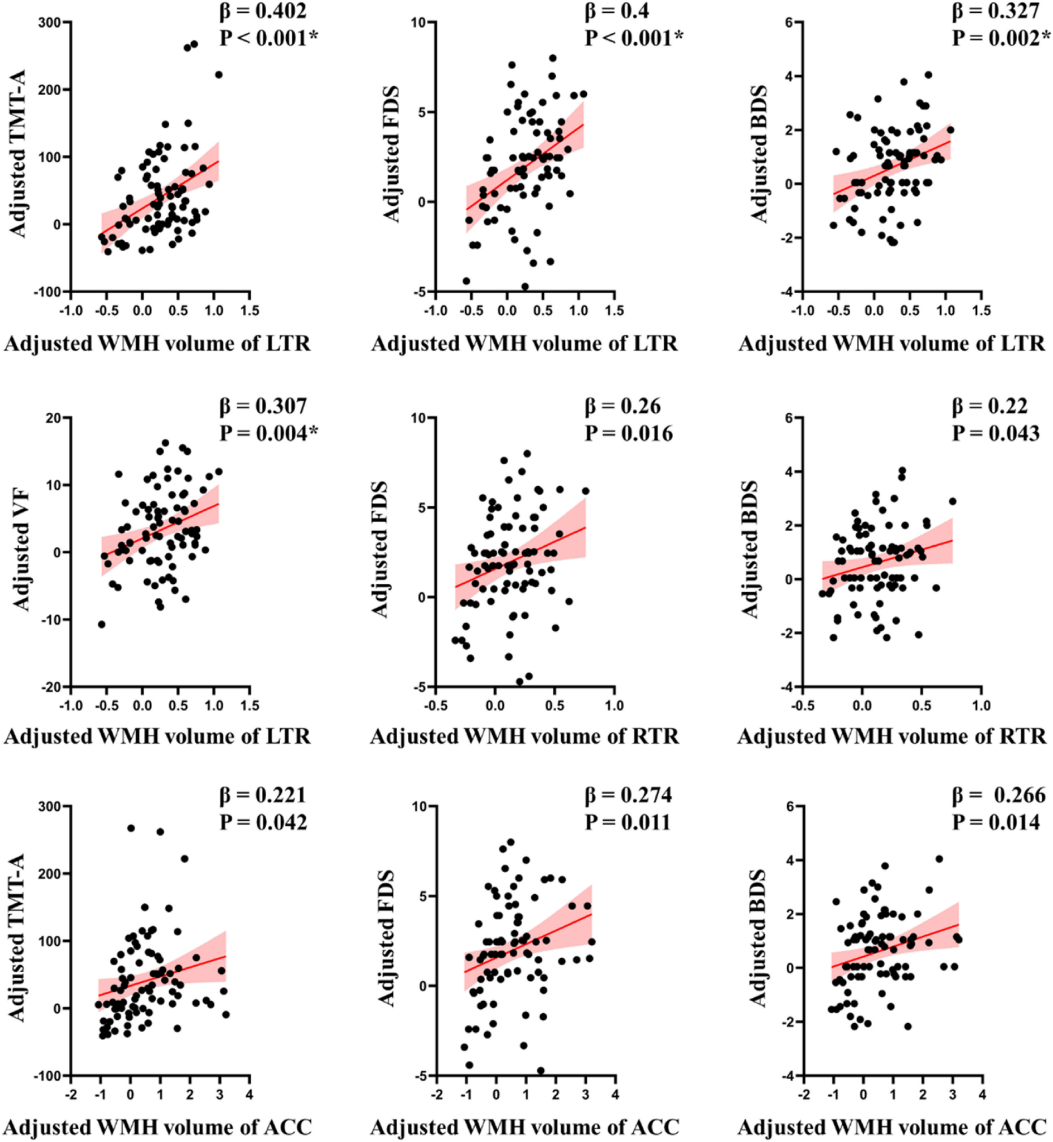
Association between the tract‐specific fiber WMH volume and cognitive assessment. Adjusted for age, sex and years of education, extent of WMH volume in the left thalamic radiation was associated with reduced information processing speed, executive function and working memory. The *p* values and *β* coefficients serve as indicators of the predictive relationship between WMH volume of six specific tract fibers and cognitive assessment in mTBI. These values were derived from regression models that were adjusted for age, sex and education. * indicated a significance of *p* < 0.05, FDR corrected. ACC, anterior corpus callosum; BDS, backward digit span; FDS, forward digit span; LTR, left thalamic radiation; RTR, right thalamic radiation; TMT‐A, trail making test A; VF, verbal fluency.

Associations between tract‐specific WMH volumes and cognitive scores obtained from the generalized linear regression modeling were shown in the Figure 5 and Table S3. Adjusted for age, sex and years of education, extent of WMH volume in the left thalamic radiation was associated with reduced information processing speed. For every 1 mL increase in WMH volume within the left thalamic radiation (8.7%, the ratio of lesion volumes to fiber tract volumes), the model predicted 65.6 s (95% CI, 33.1–98.2 s) longer completion time in the information processing speed test (*β* = 0.402, *p* < 0.001, FDR corrected). Extent of WMH volume in the left thalamic radiation was also associated with reduced executive function and working memory. For every 1 mL increase in WMH volume of the left thalamic radiation, the executive functions test increase 2.9 scores (95% CI, 1.45–4.4 scores, *β* = 0.4, *p* < 0.001, FDR corrected) and the working memory test increase 1.2 scores (95% CI, 0.44–1.96 scores, *β* = 0.327, *p* = 0.002, FDR corrected). These results suggested that increased WMH burden in the left thalamic radiation was associated with worse performance in executive function and working memory. In addition, there was a trend toward an association between tract‐specific WMH volume in the right thalamic radiation and anterior corpus callosum with impaired cognitive performances (detailed information described in the Data S1). No significant associations were observed between other tract‐specific lesion loads and cognitive ratings.

**FIGURE 6:**
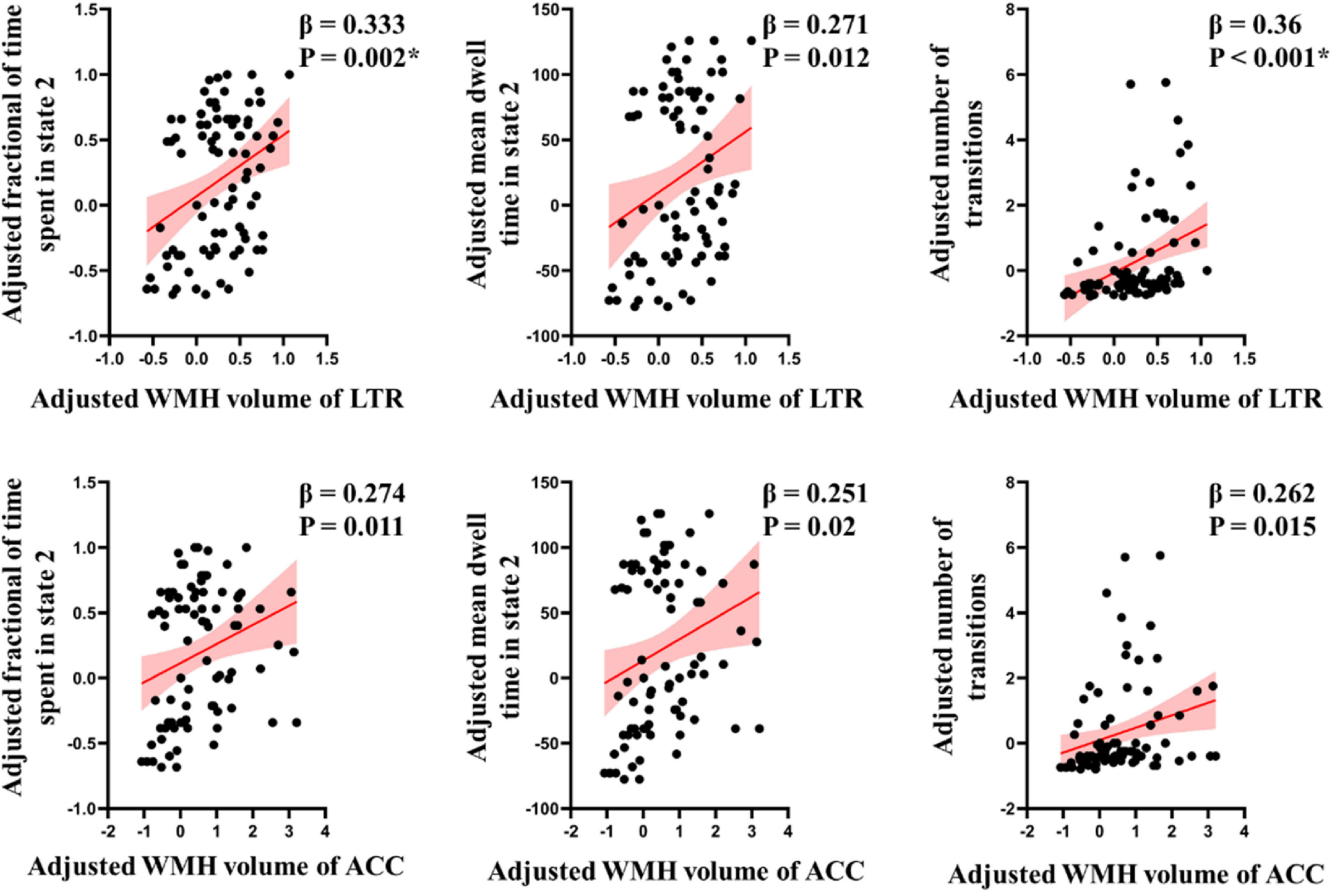
Association between the tract‐specific fiber WMH volume and brain dynamic characteristics. Adjusted for age, sex and years of education, extent of WMH volume in the left thalamic radiation was associated with longer fractional of time spent in the state 2 and increased state transitions. The *p* values and *β* coefficients serve as indicators of the predictive relationship between WMH volume of six specific tract fibers and cognitive assessment in mTBI. These values were derived from regression models that were adjusted for age, sex and education. * indicated a significance of *p* < 0.05, FDR corrected. ACC, anterior corpus callosum; LTR, left thalamic radiation.

Regression modelling adjusted for age, sex, years of education, WMH lesion burdens correlated with disrupted brain dynamics. For every 1 mL increase in WMH volume of the left thalamic radiation, the model predicted 47% (95% CI = [18–76]) longer fractional of time spent (*β* = 0.333, *p* = 0.002, FDR corrected) in the state 2. Additionally, with very 1 mL increase WMH volume of the left thalamic radiation, a 1.38‐fold increase in the state transitions (95% CI, 0.6–2.17, *β* = 0.36, *p* < 0.001, FDR corrected). There was a trend toward an association of the tract‐specific WMH volume in the right thalamic radiation and anterior corpus callosum with brain dynamics (details in the Data S1). No significant associations were observed between other tract‐specific lesion loads and brain dynamic characteristics (Figure 6 and Table S4).

Regression modelling adjusted for age, sex and years of education, brain dynamic was associated with the impaired cognitive function. For every 1% increase in fractional of time spent in the state 2, the model predicted 51.85 s (95% CI, 29.23–74.47 s) longer completion time in information processing speed test (*β* = 0.45, *p* < 0.001, FDR corrected) and the executive functions test increase 2.29 scores (95% CI, 1.29–3.3 scores, *β* = 0.445, *p* < 0.001, FDR corrected). Additionally, every 1 TR increase in mean dwell time in state 2, the model predicted 0.42 s (95% CI, 0.23–0.6 s) longer completion time in information processing speed test (*β* = 0.44, *p* < 0.001, FDR corrected) and the executive functions test increase 0.018 scores (95% CI, 0.01–0.026 scores, *β* = 0.427, *p* < 0.001, FDR corrected). No significant associations were observed between other tract fibers and brain dynamics (details in the Table S5).

## 4 Discussion

Our analysis yielded three main results. First, increased WMH lesion loads within the thalamic radiation and corpus callosum were highest among all tract fibers following mTBI, and related with altered FA within the same tracts. Second, our findings indicated significant relations between higher WMH burden and prolonged mean dwell time, along with an augmented fraction of time spent in state involved stronger internetwork connections. Third, thalamic radiation, emerged as a structural determinant for spatiotemporal brain dynamics, mediating cognitive deficits associated with WMH burdens.

Increased WMH lesion loads within the thalamic radiation and corpus callosum were highest among all tract fibers in patients with mTBI, and related with integrity loss within the same tracts. These tract‐specific WMH were regional distributed that the ratio was almost 7.25% for the thalamic radiation and 4.93% for the anterior corpus callosum. Therefore, we adopted the AFQ to measure the diffusion metrics at multiple nodes along a white matter pathway (tract) instead of merely a global mean value, which is more sensitive to detect the focal or regional integrity affected by the WMH lesion load. This method is also sensitive to characterize white matter pathology in clinically heterogeneous patients (i.e., mTBI), which are free of the implicit assumption that have a homogenous (i.e., high degree of spatial overlap) pattern of white matter abnormalities. By using the AFQ, we observed that the higher tract‐specific WMH loads contributed to the widespread increased FA in the same tract fibers, suggesting diffuse abnormalities in myelin and/or fiber axons. Specifically, inflammatory response produced by traumatic microbleeds, promote excitotoxicity, and oxidative injury giving rise to the neurotoxicity in acute mTBI, then resulting in increased FA (Jolly et al. 2020; Johnson et al. 2023). Meanwhile, patients with higher WMH lesion burdens also exhibited the increased serum inflammation level of the IL‐1β, IL‐6 and TNF‐α. These findings suggested that WMH lesion may be partly due to the inflammatory response, thereby contributed to the increased FA.

Consequently, WMH, preferentially damaging the long‐range fibers, would lead to the impaired communications among brain networks (Sharp, Scott, and Leech 2014). A large population‐based study of brain connectivity also provides the evidence that WMH‐related structural connectome disconnection can contribute to the worse cognitive functions (Langen et al. 2018). In the present study, for every 1 mL increase in WMH volume within the left thalamic radiation, the model predicted 65.6 s longer completion time in the information processing speed test. Extent of WMH volume in the thalamic radiation was also associated with reduced executive function and working memory. Taken together, these results suggested that the higher WMH load would disrupt white matter tracts, thereby affect the tract‐connected function areas and associated cognitive impairments (Sharp, Scott, and Leech 2014; Taylor et al. 2017; van der Horn et al. 2020).

Our study also provided the first evidence for the structural determinants of spatiotemporal brain dynamics in patients with mTBI. Research from the Human Connectome Project had indicated that the global geometry and topology of the whiter matter connectome is an important factor to modulate the temporal fluctuations of brain activity (Fukushima and Sporns 2020). Neurological conditions, such as the cerebral small vessel disease and stroke, also manifest that altered temporal properties of the DFC can be modulated by the structural connectome (Fu et al. 2019; Bonkhoff et al. 2020). We found two distinct connectivity states across the entire group, segregated state characterized by the sparse inter‐independent component connections, and DMN‐centered integrated state with strongly internetwork connections between DMN and other networks. The thalamic radiation linking thalamic with specific cortical areas, mainly involving DMN (Zhang et al. 2010). In our finding, its integrity loss was also accompanied with the aberrant dynamic characteristics, such as the longer mean dwell time and more fractional of time spent in the stronger internetwork connections (i.e., DMN‐centered integrated state). Of which, the longer mean dwell time implying the mTBI patients lingered more temporal duration in DMN‐centered integrated state, and the more fractional of time spent meaning the mTBI patients had higher percentage of total time spent in DMN‐centered integrated state.

These results are consistent with those obtained other neurological (Alzheimer's disease and epilepsy) and psychiatric (schizophrenia) conditions, all showed altered mean dwell time and fractional of time spent compared to controls (Liu et al. 2017; Supekar et al. 2019; Fiorenzato et al. 2019). In addition, DFC temporal properties were closely associated with cognitive performance on several domains (i.e., attention, executive, memory and visuospatial) in mTBI (Li et al. 2023). More specifically, for every 1% increase in fractional of time spent in the state 2, the model predicted 51.85 s longer completion time in information processing speed test and the executive functions test increase 2.29 scores. Increase in mean dwell time in state 2 was also associated with reduced information processing speed and executive function. Thus, our findings lead us to suggest higher fractional of time spent in states 2 and increased dwell time in state 2 were associated with cognitive decline in mTBI.

Patients with cerebrovascular disease commonly exhibit deficits in cognitive processing speed (Duering et al. 2014). It has proven to be resulted from a disruption of frontal‐subcortical neuronal circuits by WMH lesions, but the exact mechanism and underlying anatomical structures are poorly understood. In this study, the thalamic radiation was identified as the strategic white matter tract to mediate the WMH lesion injury with the dynamic brain characteristics and cognitive processing speed. Specially, every 1 mL increase in WMH volume with the left thalamic radiation was associated with a 47% increase fractional occupancies in state 2 and contributed to 65.6 s delay in completion of cognitive processing speed test. Our findings confirm and extend previous results identifying the strategic white matter tracts, with the highest predictive value for processing speed in TBI patients with small vessel lesions.

Strengths of this study were tract‐specific WMH load analysis and used AFQ to compute the diffusion metrics in pinpoint regions rather than averaged diffusion metrics along entire tract fiber. Furthermore, we applied dynamic instead of static functional connectivity because of the subtle network disruption would become more apparent in the dynamic feature of brain circuits (Fiorenzato et al. 2019; Schlemm et al. 2022; Li et al. 2023). However, there are a few limitations that should be considered. First, limited to the cross‐sectional observational research, no causal relationships can be assessed. Particularly, this study cannot test the hypothesis that brain dynamics casually mediate the tract‐specific WMH lesion on cognitive deficits in mTBI. In the further study, we would apply mediation analysis to test whether alterations in brain dynamic are on the potential causal pathway of the association between WMH volume and cognition, as it is biologically plausible that the function network is affected by WMH. The controlled treatment interventions focusing on the modulation of the WMH lesion would make such exploration possible. Second, on average, mTBI patients enrolled were only mildly affected by WMH lesions, which limit generalizability of conclusions to more severely affected populations with the exposure of TBI.

## 5 Conclusions

The paper provides the first evidence that structural determinants of spatiotemporal brain dynamics in patients with mTBI and their relevance for cognitive deficits. More specifically, the higher regional tract‐specific WMH burden can disrupt long‐range whiter matter fibers, thereby affect the tract‐connected function areas and cause the aberrant dynamic brain state. Meanwhile, the detrimental effect of larger WMH volume on cognitive function was mediated by dynamic brain state. In addition, the WMH burden of thalamic radiation is important for the dynamic brain characteristics and cognitive processing speed, which means the thalamic radiation had highest predictive value in mTBI.

## Conflicts of Interest

The authors declare no conflicts of interest.

## Supporting information


**Data S1:** Supporting Information.

## Data Availability

The data that support the findings of this study are available on request from the corresponding author. The data are not publicly available due to privacy or ethical restrictions.
