# Supplementary material for: Tract‐Specific White Matter Hyperintensities Disrupt Brain Networks and Associated With Cognitive Impairment in Mild Traumatic Brain Injury
Source: Hum Brain Mapp. 2024 Nov 29;45(17):e70050. doi: 10.1002/hbm.70050 (PMC11605479; doi:10.1002/hbm.70050)
Supplement: Supplementary file 1 — Data S1: Supporting Information. [file HBM-45-e70050-s001.docx]

***Supplementary materials***

**Materials and methods**

**DTI data** **preprocessing and automated fiber quantification**

Preprocessing of DTI data was performed using FMRIB Software Library (FSL) (https://fsl.fmrib.ox.ac.uk/fsl) software (Jenkinson et al. 2012). First, non-brain tissues were removed from the DTI data using the brain extraction tool algorithm in FSL. Next, eddy correction was carried out to correct the effects of head movement and geometric distortion caused by the eddy current. Diffusion data were visually inspected after each processing step to control obvious artifacts. Then, diffusion metrics were calculated using the DTIFIT tool in FSL, and subsequently, fractional anisotropy (FA) maps were obtained. And each participants FA image was aligned to MNI-152 standard space using non-linear registration Tool (FLIRT).

After preprocessing, we used a MATLAB‐based open source software Automated Fiber-tract Quantification (AFQ) (<https://github.com/jyeatman/AFQ>) to identify 20 white matter tracts in each participant’s brain(Yeatman et al. 2012). The identification procedure included three primary steps. Firstly, whole brain deterministic fiber tracking was performed on preprocessed tensor images using a streamline tracking algorithm with a fourth‐order Runge‐Kutta path integration method (Basser et al. 2000). The tracking algorithm starts within a white matter mask defined as voxels with FA value greater than 0.3, then the path integration procedure traces the fiber in both directions along principal diffusion axes. The tracing is terminated when the FA value becomes lower than 0.2 or the minimum angle between the last path segment and next step is greater than 30°. Secondly, fiber tract segmentation was performed using the waypoint ROI procedure described by Wakana et al (Wakana et al. 2007). The waypoint ROI sets developed at the John Hopkins Medical Institute (http://cmrm.med.jhmi.edu/) were warped from the MNI template space into individual coordinate space via non‐linear transformation. Each fiber was defined as a candidate to a particularly fiber group if it passed through two waypoint ROIs that define a specific fiber tract. Finally, fiber refinement was accomplished by comparing each candidate fiber to fiber tract probability maps develop by Hua et al (Hua et al. 2008). The fiber tract probability maps were also registered to each individual space and then candidate fibers for a particularly fiber group were assigned scores according to probability values of the voxels they pass through. If a candidate fiber does not get the highest probability score to the same fiber group, it was judged to have an aberrant trajectory and would be discarded. The identified 20 tracts were bilateral thalamic radiation, corticospinal tract, cingulum cingulate, cingulum hippocampus, inferior fronto-occipital fasciculus, inferior longitudinal fasciculus, superior longitudinal fasciculus, uncinate fasciculus, arcuate fasciculus, posterior and anterior corpus callosum (Figure. S2).

**Functional data preprocessing and** **dyna****mic functional networks analysis**

A standard functional MRI preprocessing procedure was implemented using DPABI (http://rfmri.org/dpabi) and SPM 12 (https://www.fil.ion.ucl.ac.uk/spm), including the slice-timing correction, realignment, normalization, spatial smoothing (6 mm smoothing kernel), regression of nuisance variables (24 motion parameters, white matter, global, and cerebrospinal fluid signal) and band-pass filtering (0.01 Hz < f < 0.1 Hz). Participants with motion greater than 2 mm in any direction or 2◦ of rotation during the scanning were excluded. Considering that the dynamic FC analysis may be sensitive to gross head motion effects, we also calculated the mean frame-wise displacement (FD) to scrubbed the “bad” volume with excessive head-motion (FD > 1 mm).

Our previous work has demonstrated that dysregulated brain dynamic interactions can imply more server impairments across multiple cognitive domains and explain the highly disparate outcomes among patients with mTBI (Li et al. 2023). In current study, we adopted the sliding window dynamic functional connectivity approach to construct the dynamic functional connectivity and adopted k-means clustering algorithm to investigate the temporal properties of dynamic functional connectivity states. The procedure included two primary steps: Firstly, we extracted a time series of fMRI signals from each of 227 regions of interest (ROIs) that were selected from the 264 ROIs listed in the previous studies (Power et al. 2011). The other 37 ROIs were not adopted here because they were labelled ‘Uncertain’ and did not constitute specific cortical networks. The 227 cortical ROIs were defined as 4-mm spheres around the center coordinates that were determined in the previous studies (Power et al. 2011). Based on prior studies, we then classified the ROIs into eight functionally different brain networks (Power et al. 2011). Then, we adopted the sliding window dynamic functional connectivity approach and the sliding window length of 50 TR and shifted with a step size of 1 TR were selected in the current study (Li et al. 2023). For each subject, this step resulted in 126 windows in total. In each sliding window, the time series of 227 ROIs were extracted and the Pearson’s correlated coefficient between time series of each pair of these 227 ROIs were calculated. Then we obtained a 227 $\times$ 227 correlation matrix, and a Fisher’s r-to-z transformation was applied to all the correlation matrix to improve the normality of the correlation distribution. Secondly, dynamic functional networks states analysis. In detail, we adopted a k-means clustering algorithm on all windowed functional connectivity matrices (137 subjects $\times$ 126 windows = 17,262 matrices) to assess the reoccurring functional connectivity patterns (states), as expressed by the frequency and structure of these states. We used L1 distance (Manhattan distance) function to estimate the similarity between window functional connectivity matrices, as it has been demonstrated to be an effective measure for high-dimensional data (Aggarwal, Hinneburg, and Keim 2001). Moreover, we performed a cluster validity analysis (silhouette) on the exemplars of all the subjects to estimate the optimal number of clusters. In detail, a subsampling analysis was conducted to reduce the redundancy between windows and computational demands (Allen et al. 2014). Subsampling was used to obtain the reliable results from the whole windows without biasing group clusters (Allen et al. 2014), and thus the reproducibility of functional connectivity states was established using replications on bootstrap resampling and split-half sample methods. Based on the silhouette criterion of cluster validity index, the optimal number of clusters was determined as k = 2. In addition, the optimal number of clusters k was 2 by estimated Calinski-Harabasz coefficients and Davies-Bouldin coefficients on all windows of all subjects varying k from 2 to 6. Additionally, we investigated the temporal properties of dynamic functional connectivity states by computing the mean dwell time and fractional of time spent in each state, as well as the number of transitions from one state to another. Specifically, the mean dwell time was the average number of consecutive windows in one state. The fractional of time spent was measured as the proportion of all windows in each state. The number of transitions was defined as the total number of state switches between consecutive windows. Figure S3 showed the analysis pipeline and the detailed processing of the dynamic functional network analysis.

**Results**

**Associations between WMH volume,** **cognitive, and brain dynamics**

In our current study, we explored the potential links between the WMH volume in the tract fibers and neuropsychological assessments, as well as the temporal properties of dynamic functional connection. Associations between the tract-specific WMH volume and cognitive scores obtained from generalized linear regression modeling are shown in Figure 6 and Supplementary Table S3. Adjusted for age, sex and education, extent of WMH volume in LTR was associated with IPS. For every 1 ml increased in WMH volume of LTR (8.7%, the ratio of lesion volumes to tract volumes), the model predicted increased 65.6 seconds (95% CI [33.1 to 98.2]) completion time in TMT-A (β = 0.402, P < 0.001, FDR corrected). In addition, extent of WMH volume in LTR was associated with executive functions and working memory. For every 1 ml increased in WMH volume of LTR, the FDS test increased 2.9 scores (95% CI [1.45 to 4.4], β = 0.4, P < 0.001, FDR corrected) and the BDS test increased 1.2 scores (95% CI [0.44 to 1.96], β = 0.327, P = 0.002, FDR corrected). Similarly, with very 1 ml increased WMH volume of LTR, the VF test was increased 4.8 scores (95% CI [1.56 to 8.05], β = 0.307, P = 0.004, FDR corrected). There was a trend toward an association between tract-specific WMH volume in RTR and ACC with reduced cognitive scores. We also found that for every 1ml increased in WMH volume of RTR (8.0 %, the ratio of lesion volumes to tract volumes), the model predicted increased 3.04 scores (95% CI [0.58 to 5.5]) in FDS test (β = 0.26, P = 0.016) and increased 1.3 scores (95% CI [0.04 to 2.56]) in BDS test (β = 0.22, P = 0.043). The WMH volume of ACC correlated with the cognitive scores. For every 1ml increased in WMH volume of ACC (19.4 %, the ratio of lesion volumes to tract volumes), the model predicted increased 13.64 seconds (95% CI [0.48 to 26.8]) completion time in TMT-A (β = 0.22, P < 0.042). With very 1 ml increased WMH volume of ACC, the FDS test increased 0.76 scores (95% CI [0.18 to 1.3], β = 0.27, P < 0.011) and the BDS test increased 0.37 scores (95% CI [0.077 to 0.66], β = 0.27, P = 0.014) (Supplementary Table S3). No significant associations were observed between other tract fibers and cognitive scores. No significant associations were observed between other tract fibers and cognitive scores (Supplementary Table S3).

In the generalized linear regression models, WMH volume in the tract fibers correlated with temporal properties of dynamic functional connection. For every 1 ml increased in WMH volume of LTR, the model predicted increased 0.47 % (95% CI [0.18 to 0.76]) in fractional of time spent in state 2 (β = 0.333, P = 0.002, FDR corrected) and increased 46.37 TR (95% CI [10.37 to 82.37]) in mean dwell time in state 2 (β = 0.271, P = 0.012). In addition, with very 1 ml increased WMH volume of LTR, the number of transitions increased 1.38 times (95% CI [0.6 to 2.17], β = 0.36, P = 0.001, FDR corrected). There was a trend toward an association between tract-specific WMH volume in RTR and ACC with brain dynamic. We also found that for every 1 ml increased in WMH volume of ACC, the model predicted increased 0.15 % (95% CI [0.034 to 0.258]) in fractional of time spent in state 2 (β = 0.274, P = 0.011) and increased 16.32 TR (95% CI [2.6 to 30.04]) in mean dwell time in state 2 (β = 0.251, P = 0.02). With very 1 ml increased WMH volume of ACC the number of transitions increased 0.38 times (95% CI [0.075 to 0.688], β = 0.262, P = 0.015). No significant associations were observed between other tract fibers and temporal properties of dynamic functional connection (Supplementary Table S4).

Table S1. Serum inflammation cytokine levels for patients with mTBI and HC

| Serum inflammation cytokines | mTBI (n =62) | HC (n = 34) | p Value |
| --- | --- | --- | --- |
| IL-1β | 0.83(1.23) | 0.13(1.32) | **0.011** |
| IL-6 | 0.87(1.85) | -0.38(0.83) | **< 0.001** |
| IL-8 | 2.69(5.65) | 1.10(6.02) | 0.202 |
| IL-10 | 0.09(0.55) | 0.16(0.50) | 0.557 |
| TNF-α | 0.76(1.57) | 0.25(1.69) | 0.141 |

Two sample T test was run to test between-group differences (mTBI and HC).

Table S2. Serum inflammation cytokine levels for patients with sWMH, mWMH and HC

| Serum inflammation cytokines | mTBI (sWMH)  (n = 18) | mTBI (mWMH)  (n = 44) | HC  (n = 34) |  | ANOVA |  | Post hoc | | |
| --- | --- | --- | --- | --- | --- | --- | --- | --- | --- |
|  |  |  |  |  | p Value |  | p1Value | p2Value | p3Value |
| IL-1β | 1.10(1.38) | 0.71(1.16) | 0.13(1.32) |  | **0.023** |  | 0.823 | **0.029** | 0.138 |
| IL-6 | 1.01(1.71) | 0.81(1.91) | -0.38(0.83) |  | **0.001** |  | 0.99 | **0.009** | **0.004** |
| IL-8 | 4.52(6.50) | 1.93(5.16) | 1.10(6.02) |  | 0.124 |  | 0.332 | 0.130 | 0.99 |
| IL-10 | 0.02(0.42) | 0.12(0.59) | 0.16(0.50) |  | 0.663 |  | 0.99 | 0.99 | 0.99 |
| TNF-α | 1.42(1.69) | 0.49(1.45) | 0.25(1.68) |  | 0.041 |  | 0.120 | **0.040** | 0.99 |

One-way ANOVA was run to test between-group differences (sWMH, mWHM and HC).

Table S3. Association between WMH volume of six specific tract fibers and cognitive assessment.

| WMH volume (ml) |  | TMT_A | | |  | DSC | | |  | FDS | | |  | BDS | | |  | VF | | |
| --- | --- | --- | --- | --- | --- | --- | --- | --- | --- | --- | --- | --- | --- | --- | --- | --- | --- | --- | --- | --- |
|  |  | B (95% CI) | β | P |  | B (95% CI) | β | P |  | B (95% CI) | β | P |  | B (95% CI) | β | P |  | B (95% CI) | β | P |
| LTR |  | 65.6 (33.1 to 98.2) | 0.402 | **<0.001*** |  | 4.54 (-2.2 to 11.2) | 0.146 | 0.182 |  | 2.9 (1.45 to 4.4) | 0.4 | **<0.001*** |  | 1.2 (0.44 to 1.96) | 0.327 | **0.002*** |  | 4.8 (1.56 to 8.05) | 0.307 | **0.004*** |
| RTR |  | 75.2 (20.2 to 130.1) | 0.286 | 0.08 |  | 0.856 (-10.1 to 11.8) | 0.017 | 0.876 |  | 3.04 (0.58 to 5.5) | 0.26 | **0.016** |  | 1.3 (0.04 to 2.56) | 0.22 | **0.043** |  | 4.81 (-0.58 to 10.21) | 0..191 | 0.08 |
| LCC |  | 152.8 (-96.1 to 401.7) | 0.133 | 0.226 |  | -18.7 (-66.3 to 28.9) | -0.085 | 0.437 |  | 4.01 (-7.1 to 15.1) | 0.078 | 0.476 |  | 1.8 (-3.8 to 7.439) | 0.07 | 0.527 |  | 12.87 (-11.04 to 36.77) | 0.117 | 0.287 |
| RCC |  | 128.7 (-356.4 to 613.9) | 0.058 | 0.599 |  | -5.0 (-97.49 to 87.4) | -0.012 | 0.914 |  | 15.02 (-6.352 to 36.4) | 0.152 | 0.166 |  | 6.5 (-4.4 to 17.3) | 0.129 | 0.239 |  | -2.57 (-49.14 to 44) | -0.012 | 0.913 |
| ACC |  | 13.64 (0.479 to 26.8) | 0.221 | **0.042** |  | -0.44 (-3.0 to 2.1) | -0.038 | 0.732 |  | 0.76 (0.18 to 1.3) | 0.274 | **0.011** |  | 0.37 (0.077 to 0.66) | 0.266 | **0.014** |  | 0.83 (-0.45 to 2.11) | 0.14 | 0.203 |
| PCC |  | -11.5 (-167.4 to 144.4) | -0.016 | 0.884 |  | -4.6 (-34.2 to 25.1) | -0.034 | 0.759 |  | -3.5 (-10.38 to 3.4) | -0.11 | 0.318 |  | -1.5 (-4.9 to 1.9) | -0.093 | 0.395 |  | -5.63 (-20.52 to 9.26) | -0.082 | 0.454 |

The P-values and β coefficients serve as indicators of the predictive relationship between WMH volume of six specific tract fibers and cognitive assessment in mTBI. These values were derived from regression models that were adjusted for age, sex and education. To account for the potential for multiple comparisons, the P-values underwent adjustment using the FDR method, with a corrected significance threshold of P < 0.05. * FDR corrected.

Abbreviations: LTR, left thalamic radiation; RTR, right thalamic radiation; LCC, left cingulum cingulate; RCC, right cingulum cingulate; ACC, anterior corpus callosum; PCC, posterior corpus callosum. TMT-A, trail making test A; DSC, Digital Symbol Coding score; FDS, Forward digit span; BDS, Backward digit span; VF, verbal fluency.

Table S4. Association between WMH volume of six specific tract fibers and brain dynamics.

| WMH volume (ml) |  | Fractional of time spent in state 1 | | |  | Fractional of time spent in state 2 | | |  | Mean dwell time in state 1 | | |  | Mean dwell time in state 2 | | |  | Number of transitions | | | |
| --- | --- | --- | --- | --- | --- | --- | --- | --- | --- | --- | --- | --- | --- | --- | --- | --- | --- | --- | --- | --- | --- |
|  |  | B (95% CI) | β | P |  | B (95% CI) | β | P |  | B (95% CI) | β | P |  | B (95% CI) | β | P |  | B (95% CI) | β | P |  |
| LTR |  | -0.014(-0.28 to 0.25) | -0.012 | 0.917 |  | 0.47(0.18 to 0.76) | 0.333 | **0.002*** |  | -14.25(-46.82 to 18.32) | -0.095 | 0.387 |  | 46.37(10.37 to 82.37) | 0.271 | **0.012** |  | 1.38(0.6 to 2.17) | 0.36 | **0.001*** |  |
| RTR |  | 0.08(-0.34 to 0.5) | 0.041 | 0.375 |  | 0.436(0.49 to 0.92) | 0.192 | 0.078 |  | 3.25(-49.44 to 55.95) | 0.013 | 0.903 |  | 46.72(-12.64 to 106.09) | 0.169 | 0.121 |  | 1.03(-0.298 to 2.36) | 0.167 | 0.127 |  |
| LCC |  | 0.2(-2.06 to 1.65) | -0.024 | 0.83 |  | 0.878(-1.281 to 3.04) | 0.088 | 0.421 |  | -16.67(-247.39 to 214.06) | -0.016 | 0.886 |  | 114.54(-148.02 to 377.09) | 0.095 | 0.388 |  | 0.342(-5.571 to 6.25) | 0.013 | 0.909 |  |
| RCC |  | -1.62(-5.2 to 1.95) | -0.099 | 0.369 |  | 2.81(-1.35 to 6.96) | 0.146 | 0.183 |  | -210.5(-654.71 to 233.7) | -0.103 | 0.349 |  | 322.85(-182.68 to 828.38) | 0.138 | 0.208 |  | 6.53(-4.83 to 17.88) | 0.125 | 0.256 |  |
| ACC |  | -0.024(-0.124 to 0.75) | -0.053 | 0.631 |  | 0.146(0.034 to 0.258) | 0.274 | **0.011** |  | -6.76(-19.07 to 5.55) | -0.119 | 0.278 |  | 16.32(2.6 to 30.04) | 0.251 | **0.02** |  | 0.38(0.075 to 0.688) | 0.262 | **0.015** |  |
| PCC |  | -0.94(-2.08 to 0.19) | -0.178 | 0.102 |  | 0.81(-0.523 to 2.15) | 0.132 | 0.23 |  | -136.66(-276.78 to 3.46) | -2.08 | 0.056 |  | 99.4(-62.89 to 261.71) | 0.133 | 0.227 |  | 1.43(-2.23 to 5.07) | 0.085 | 0.44 |  |

The P-values and β coefficients serve as indicators of the predictive relationship between WMH volume of six specific tract fibers and brain dynamics in mTBI. These values were derived from regression models that were adjusted for age, sex and education. To account for the potential for multiple comparisons, the P-values underwent adjustment using the FDR method, with a corrected significance threshold of P < 0.05. * FDR corrected.

Abbreviations: LTR, left thalamic radiation; RTR, right thalamic radiation; LCC, left cingulum cingulate; RCC, right cingulum cingulate; ACC, anterior corpus callosum; PCC, posterior corpus callosum.

Table S5. Association between brain dynamics and cognitive assessment.

| Brain  dynamics |  | TMT_A | | |  | DSC | | |  | FDS | | |  | BDS | | |  | VF | | |
| --- | --- | --- | --- | --- | --- | --- | --- | --- | --- | --- | --- | --- | --- | --- | --- | --- | --- | --- | --- | --- |
|  |  | B (95% CI) | β | P |  | B (95% CI) | β | P |  | B (95% CI) | β | P |  | B (95% CI) | β | P |  | B (95% CI) | β | P |
| Fractional of time spent in state 1 |  | 8.33(-21.15 to 37.81) | 0.062 | 0.58 |  | 5.01(-0.49 to 10.53) | 0.195 | 0.074 |  | 0.912(-0.39 to 2.21) | 0.151 | 0.166 |  | 0.355(-0.305 to 1.02) | 0.117 | 0.288 |  | 2.21(-0.58 to 5.003) | 0.171 | 0.118 |
| Fractional of time spent in state 2 |  | 51.85(29.23 to 74.47) | 0.45 | **<0.001*** |  | 1.99(-2.79 to 6.79) | 0.091 | 0.41 |  | 2.29(1.29 to 3.3) | 0.445 | **<0.001*** |  | 0.71(0.16 to 1.26) | 0.272 | **0.012** |  | 3.43(1.12 to 5.73) | 0.31 | **0.004*** |
| Mean dwell time in state 1 |  | 0.056(-0.18 to 0.29) | 0.051 | 0.64 |  | 0.038(-0.007 to 0.082) | 0.182 | 0.095 |  | 0.005(-0.005 to 0.016) | 0.111 | 0.314 |  | 0.002(-0.003 to 0.008) | 0.091 | 0.407 |  | 0.014(-0.01 to 0.37) | 0.14 | 0.21 |
| Mean dwell time in state 2 |  | 0.42(0.23 to 0.6) | 0.44 | **<0.001*** |  | 0.009(-0.031 to 0.48) | 0.049 | 0.659 |  | 0.018(0.01 to 0.026) | 0.427 | **<0.001*** |  | 0.005(0.001 to 0.01) | 0.247 | **0.023** |  | 0.025(0.006 to 0.044) | 0.271 | **0.012** |
| Number of transitions |  | 4.49(-4.73 to 13.71) | 0.11 | 0.34 |  | 1.06(-0.69 to 2.81) | 0.132 | 0.23 |  | 0.382(-0.022 to 0.787) | 0.202 | 0.063 |  | 0.15(-0.056 to 0.356) | 0.157 | 0.152 |  | 0.851(-0.018 to1.72) | 0.209 | 0.055 |

The P-values and β coefficients serve as indicators of the predictive relationship between brain dynamics and cognitive assessment in mTBI. These values were derived from regression models that were adjusted for age, sex and education. To account for the potential for multiple comparisons, the P-values underwent adjustment using the FDR, with a corrected significance threshold of P < 0.05. * FDR corrected.

Abbreviations: TMT-A, trail making test A; DSC, Digital Symbol Coding score; FDS, Forward digit span; BDS, Backward digit span; VF, verbal fluency.


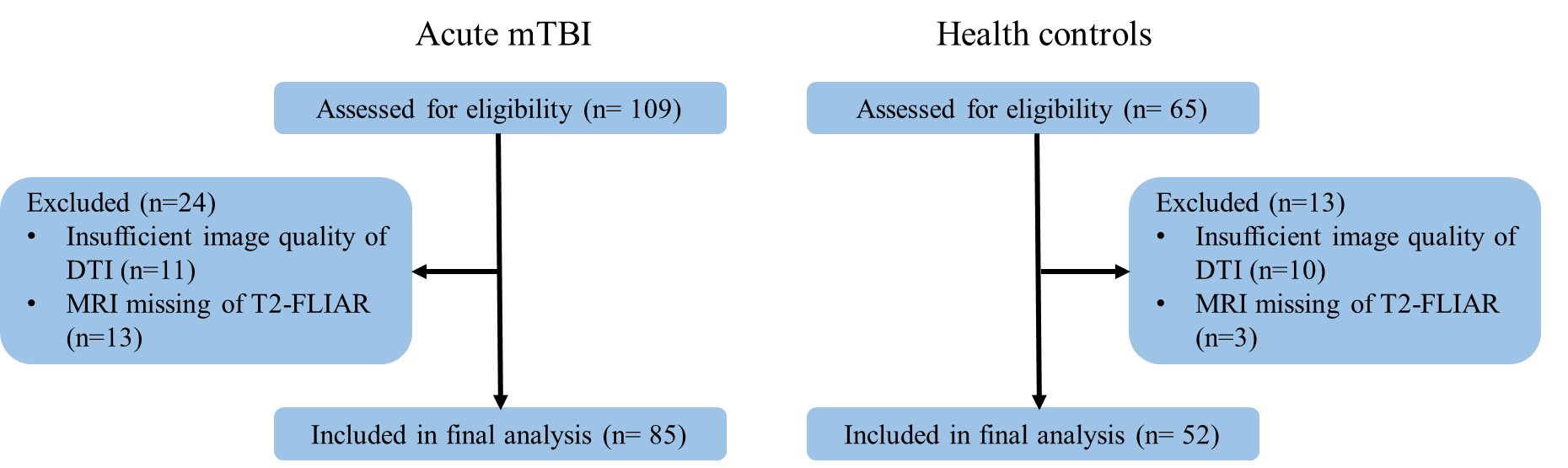


Figure S1. Flow of participant enrollment.


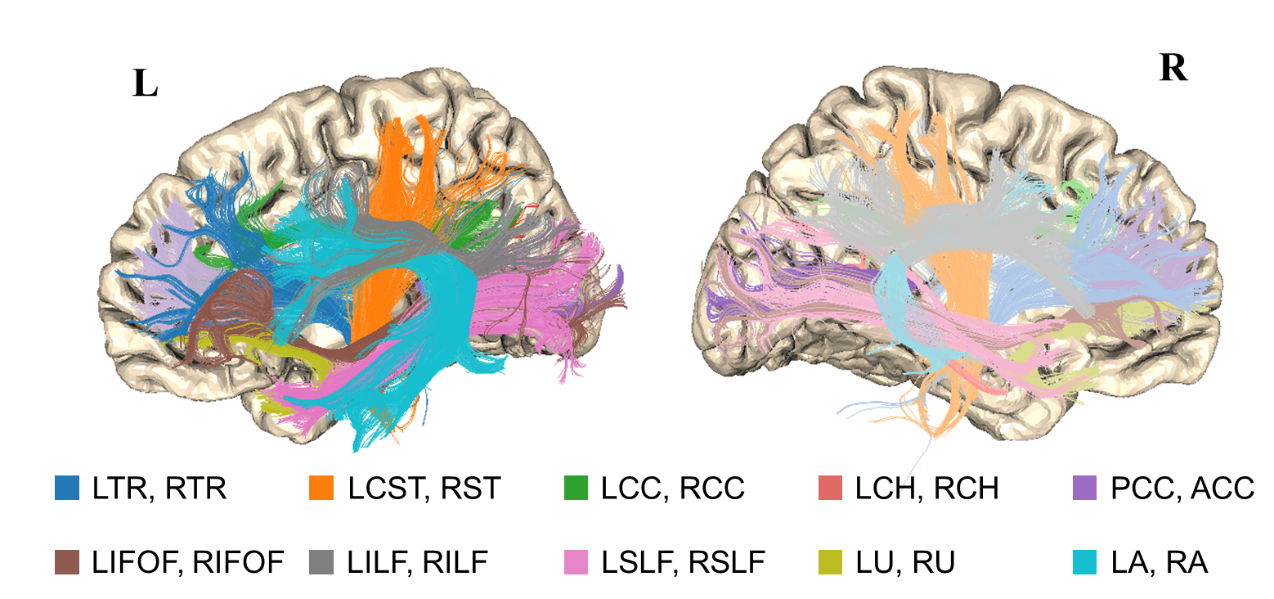


Figure S2. Fiber tracts identification result. Twenty main fiber tracts were identified by Automatic Fiber Quantification.

Abbreviations: LTR, left thalamic radiation; RTR, right thalamic radiation; LCST, left corticospinal tract; RCST, right corticospinal tract; LCC, left cingulum cingulate; RCC, right cingulum cingulate; LCH, left cingulum hippocampus; RCH, right cingulum hippocampus; LIFOF, left inferior fronto-occipital fasciculus; RIFOF, right inferior fronto-occipital fasciculus; LILF, left inferior longitudinal fasciculus; RILF, right inferior longitudinal fasciculus; LSLF, left superior longitudinal fasciculus; RSLF, right superior longitudinal fasciculus; LUF, left uncinate fasciculus; RUF, right uncinate fasciculus; LAF, left arcuate fasciculus; RAF, right arcuate fasciculus; ACC, anterior corpus callosum; PCC, posterior corpus callosum.


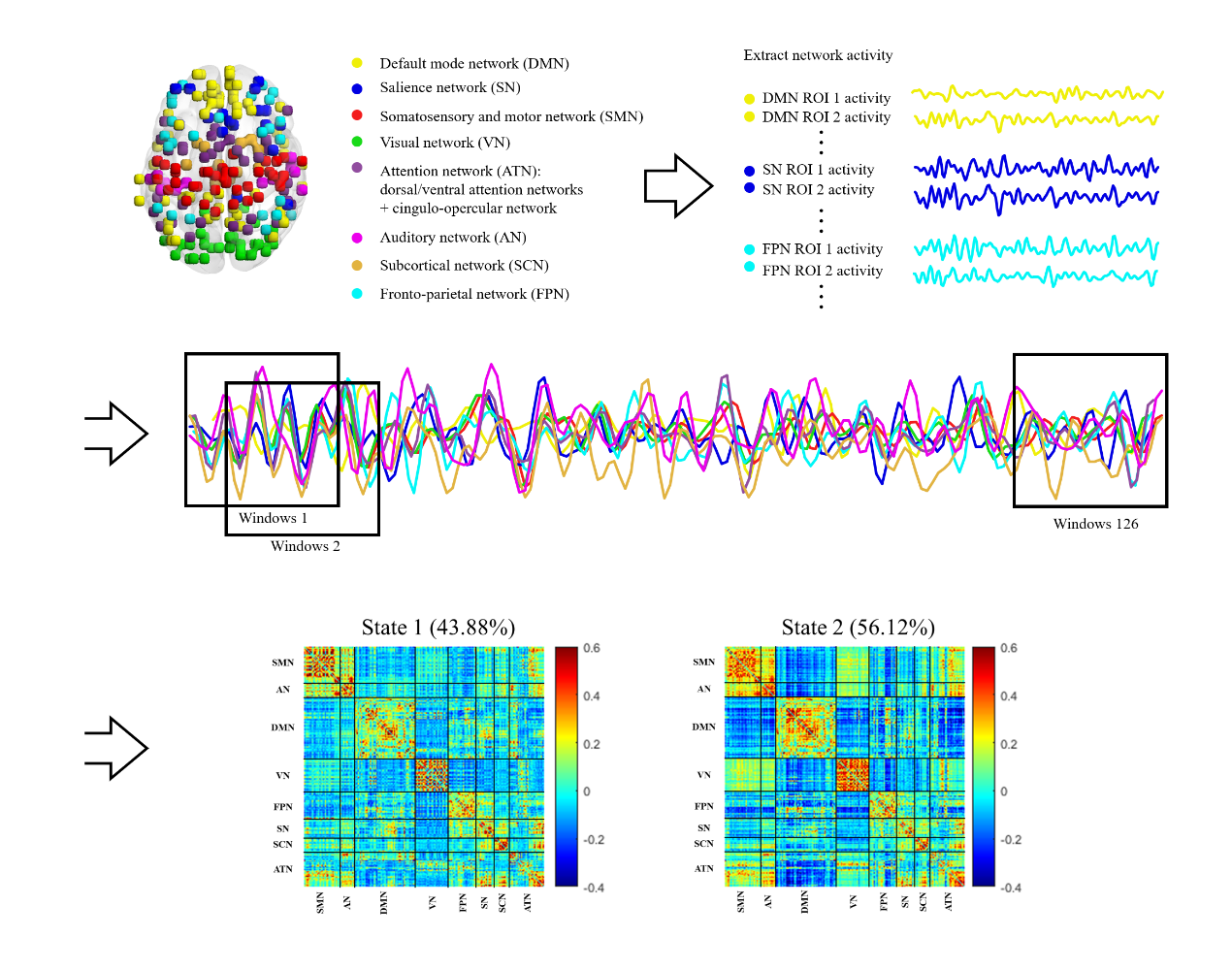


Figure S3. Procedures of dynamic functional connectivity analysis.


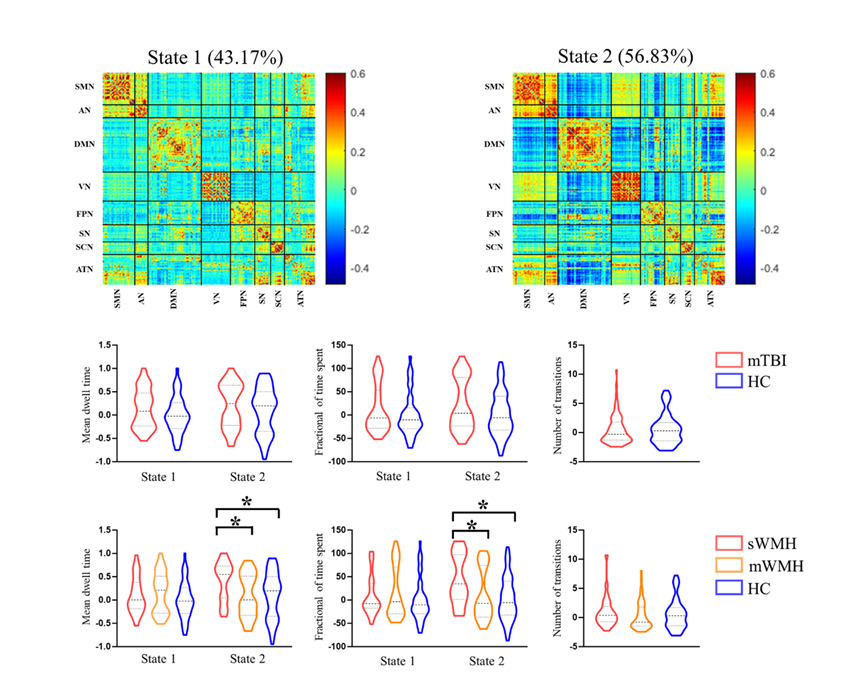


Figure S4. Sliding window length of 30 TR and shifted with a step size of 1 TR. Temporal properties of dynamic FC states for the mTBI and HC groups and temporal properties of dynamic FC states for the sWMH subgroup, mWMH subgroup and HC groups. * indicated a significance of P < 0.05, FDR corrected. Abbreviations: mTBI, mild traumatic brain injury; WMH, white matter hyperintensities; sWMH, severe WMH load group; mWMH, mild WMH load group; HC, healthy controls


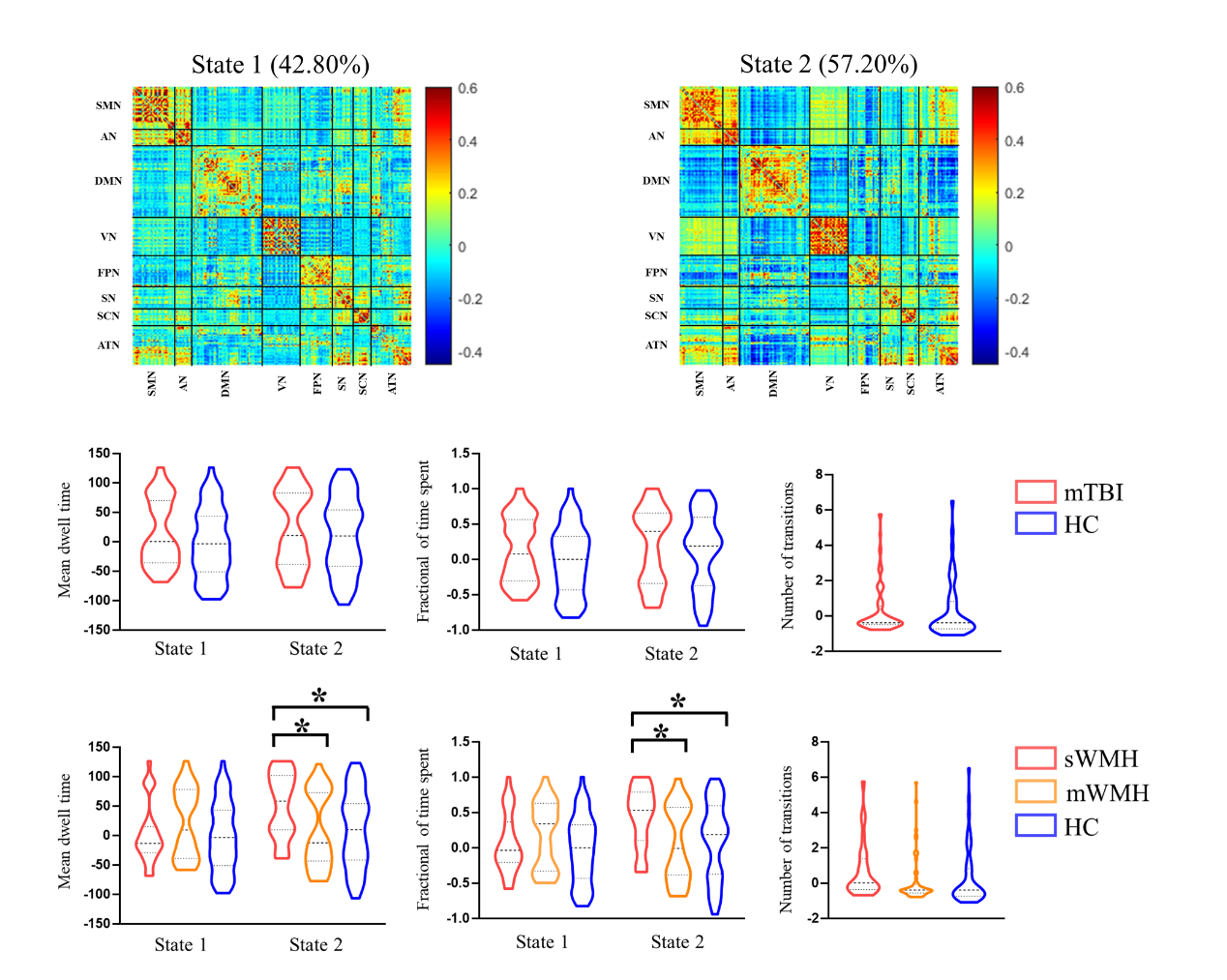


Figure S5. Sliding window length of 30 TR and shifted with a step size of 1 TR. Temporal properties of dynamic FC states for the mTBI and HC groups and temporal properties of dynamic FC states for the sWMH subgroup, mWMH subgroup and HC groups. * indicated a significance of P < 0.05, FDR corrected. Abbreviations: mTBI, mild traumatic brain injury; WMH, white matter hyperintensities; sWMH, severe WMH load group; mWMH, mild WMH load group; HC, healthy controls

Reference

Aggarwal, C. C., A. Hinneburg, and D. A. Keim. 2001. 'On the surprising behavior of distance metrics in high dimensional space', *Database Theory - Icdt 2001, Proceedings*, 1973: 420-34.

Allen, E. A., E. Damaraju, S. M. Plis, E. B. Erhardt, T. Eichele, and V. D. Calhoun. 2014. 'Tracking Whole-Brain Connectivity Dynamics in the Resting State', *Cerebral Cortex*, 24: 663-76.

Basser, P. J., S. Pajevic, C. Pierpaoli, J. Duda, and A. Aldroubi. 2000. 'In vivo fiber tractography using DT-MRI data', *Magnetic Resonance in Medicine*, 44: 625-32.

Holm, L., J. D. Cassidy, L. J. Carroll, and J. Borg. 2005. 'Summary of the WHO collaborating centre for neurotrauma task force on mild traumatic brain injury', *Journal of Rehabilitation Medicine*, 37: 137-41.

Hua, K., J. Y. Zhang, S. Wakana, H. Y. Jiang, X. Li, D. S. Reich, P. A. Calabresi, J. J. Pekar, P. C. M. van Zijl, and S. Mori. 2008. 'Tract probability maps in stereotaxic spaces: Analyses of white matter anatomy and tract-specific quantification', *Neuroimage*, 39: 336-47.

Jenkinson, M., C. F. Beckmann, T. E. Behrens, M. W. Woolrich, and S. M. Smith. 2012. 'Fsl', *Neuroimage*, 62: 782-90.

Li, X., X. Y. Jia, Y. L. Liu, G. H. Bai, Y. Z. Pan, Q. Y. Ji, Z. Y. Mo, W. P. Zhao, Y. X. Wei, S. Wang, B. Yin, J. Zhang, and L. J. Bai. 2023. 'Brain dynamics in triple-network interactions and its relation to multiple cognitive impairments in mild traumatic brain injury', *Cerebral Cortex*, 33: 6620-32.

Power, J. D., A. L. Cohen, S. M. Nelson, G. S. Wig, K. A. Barnes, J. A. Church, A. C. Vogel, T. O. Laumann, F. M. Miezin, B. L. Schlaggar, and S. E. Petersen. 2011. 'Functional Network Organization of the Human Brain', *Neuron*, 72: 665-78.

Wakana, S., A. Caprihan, M. M. Panzenboeck, J. H. Fallon, M. Perry, R. L. Gollub, K. G. Hua, J. Y. Zhang, H. Y. Jiang, P. Dubey, A. Blitz, P. van Zijl, and S. Mori. 2007. 'Reproducibility of quantitative tractography methods applied to cerebral white matter', *Neuroimage*, 36: 630-44.

Yeatman, J. D., R. F. Dougherty, N. J. Myall, B. A. Wandell, and H. M. Feldman. 2012. 'Tract Profiles of White Matter Properties: Automating Fiber-Tract Quantification', *Plos One*, 7.
